# A Markov-model simulation of IVF programs for PCOS patients indicates that coupling myo-Inositol with rFSH is cost-effective for the Italian Health System

**DOI:** 10.1038/s41598-023-44055-0

**Published:** 2023-10-18

**Authors:** Ariel Beresniak, Michele Russo, Gianpiero Forte, Antonio Simone Laganà, Mario Montanino Oliva, Cesare Aragona, Vito Chiantera, Vittorio Unfer

**Affiliations:** 1 Data Mining International SA, Geneva, Switzerland; 2 R&D Department, Lo.Li. Pharma, 00156 Rome, Italy; 3 The Experts Group on Inositol in Basic and Clinical Research (EGOI), 00161 Rome, Italy; 4 Unit of Obstetrics and Gynecology, “Paolo Giaccone” Hospital, 90127 Palermo, Italy; 5 Department of Health Promotion, Mother and Child Care, Internal Medicine and Medical Specialties (PROMISE), University of Palermo, 90127 Palermo, Italy; 6 Department of Obstetrics and Gynecology, Santo Spirito Hospital, 00193 Rome, Italy; 7 Systems Biology Group, Rome, Italy; 8 Unit of Gynecologic Oncology, National Cancer Institute-IRCCS-Fondazione “G. Pascale”, 80131 Naples, Italy; 9 UniCamillus-Saint Camillus International University of Health Sciences, Rome, Italy

**Keywords:** Health care economics, Scientific data

## Abstract

Accumulating evidence suggests that oral supplementation with myo-Inositol (myo-Ins) is able to reduce the amount of gonadotropins and days of controlled ovarian hyperstimulation (COS) necessary to achieve adequate oocyte maturation in assisted reproduction technology (ART) protocols, particularly in women affected by polycystic ovary syndrome (PCOS). We used computational calculations based on simulation modellings. We simulated in vitro fertilization (IVF) procedures—with or without intracytoplasmic sperm injection (ICSI)—with 100,000 virtual patients, accounting for all the stages of the entire IVF procedure. A Monte Carlo technique was used to account for data uncertainty and to generate the outcome distribution at each stage. We considered virtual patients with PCOS undergoing IVF cycles to achieve pregnancy. Computational data were retrieved from clinical experience and published data. We investigated three parameters related to ART protocols: cost of single procedure; efficacy to achieve ongoing pregnancy at 12 gestational weeks; overall cost per single pregnancy. The administration of oral myo-Ins during COH protocols, compared to the standard COH with recombinant Follicle Stimulating Hormone (rFSH) only, may be considered a potential strategy to reduce costs of ART for the Italian Health System.

## Introduction

Difficulties in naturally achieving pregnancy represent a major healthcare problem that affects an increasing number of couples, which often request Assisted Reproductive Technology (ART): indeed, the last official data collection of the number of ART procedures published on the International Committee for Monitoring Assisted Reproductive Technologies (ICMART) reports 1,955,908 cycles in 79 countries performed in 2017, with a 20.1% increase in cycles from 2014^1^. More recent data confirmed such trend, despite the difficulties raised with the Covid-19 pandemics^2^.

Currently, the estimated global number of ART cycles is close to 2.6 million per year (ICMART 2021), with approximately 500,000 babies born through these techniques^1^. Specifically, in vitro fertilization (IVF), which can be performed with Intracytoplasmic Sperm Injection (ICSI), represents the most common ART procedure, accounting for about 70% of all treatments worldwide. Although this percentage may vary between countries, the outcome rates with IVF techniques are quite similar^3^. Even if the prevalence of infertility among developed countries is similar, local differences in ART and embryo transfer practices still exist, implying variability in funding, regulatory environments, and sociocultural norms^4^.

Some of the costs of ART protocols are usually covered by government-funded programs and/or third-party payers, with variable contributions. In the latest (2019) global survey on ART treatments and policies by the International Federation of Fertility Societies^5^, only 40 (47%) out of 85 countries who provided details on the insurance coverage mentioned any type of financial support for ART treatments. Those differences directly reflect the number of couples who can access to treatment^6^.

The Italian Health System covers the expenses for the treatments carried out in the public structures, against a flat-rate contribution that slightly vary among the Italian regions^7^. Moreover, the national health system entirely covers the costs of the gonadotropins—including the recombinant Follicular Stimulating Hormone (rFSH)—used for ovarian stimulation within ART protocols, regardless of whether they are carried out in public or private clinics^8^.

According to the latest data from the Italian ministry of health, 97,509 ART cycles were started in Italy in 2018^9^, leading to an increasing economic impact that weights on the national health system. However, the last number of cycles registered in 2020 in Italy was equal to 80,099, with a slightly decrease of ART procedures caused by the Covid-19 pandemic^10^.

As all ART protocols in general encompass an initial step of controlled ovarian hyperstimulation (COH), finding a simple, safe, and cost-effective approach is pivotal to increase the quality of treatments in assisted reproduction, improving pregnancy rates while reducing the associated costs^11^. In this scenario, a meta-analysis of randomized controlled trials found that oral supplementation myo-Inositol (myo-Ins) in women presenting PCOS reduces the amount of gonadotropins and length of ovarian stimulation necessary to achieve adequate ovarian maturation in women undergoing COH for IVF^12^; on a further extent, these elements may suggest a reduced risk of ovarian hyperstimulation syndrome (OHSS) in this population, a complication with potentially severe detrimental outcomes^13^. Additionally, available data highlight that myo-Ins increases the number of good quality oocytes and, potentially, the clinical pregnancy rate as well^14^.

In this paper we report the results of an economic evaluation of IVF protocols by comparing two different ovarian stimulation strategies (namely, with or without myo-Ins), using a computational model specifically developed to analyze the costs of fertility programs. In detail, the aim of the study was to evaluate the outcomes and the costs of IVF cycles performed in PCOS individuals, where the stimulation with recombinant Follice Stimulating Hormone (rFSH) is associated with myo-Ins oral supplementation compared with standard stimulation protocols with rFSH only. The Monte Carlo method was used to simulate IVF procedures, accounting for all the stages of IVF protocol, starting from the ovarian stimulation and encompassing up to three subsequent cycles per patient. The number of virtual patients used for the simulation is in line the current number of ART cycles carried out in Italy each year, in order to reflect a real-world scenario.

## Materials and methods

### Study population

The baseline parameters of the population evaluated for the study are not present since the patients undergoing IVF are virtual subjects. We carried out a virtual simulation on IVF cycles basing on the data retrieved from the literature considered for the transition percentages.

No experiments on humans were performed and human tissue samples were not used. Accordingly, no relevant guidelines and regulations were breached for the present study, and indication of licensing ethical committee is not necessary.

In order to carry out the IVF cycles simulation, we considered a population of PCOS subjects diagnosed according to the Rotterdam criteria, < 40 years old, BMI < 30 kg/m^2^, normal Prolactin levels (range 5–25 ng/ml), and normal uterine cavity, absence of tubal, uterine, genetics causes of infertility.

The patients not considered for this simulation were those presenting obesity, other medical conditions causing ovulatory disorders, hyperinsulinemia, hyperprolactinemia, or thyroidal disorders, adrenal hyperplasia or Cushing syndrome, taken, at least in the previous six months, oral contraceptives, antiandrogens or any drug that could influence hormonal metabolism.

The virtual population was defined using representative PCOS patients which most commonly appear within IVF clinical practice. Furthermore, other issues that may be involved in the occurrence of typical PCOS features were excluded from the patient subset to agree with the current European Society of Human Reproduction and Embryology (ESHRE) diagnosis guidelines for PCOS selection^15^.

### Analytical setting

Decision analyses simulation models have been developed to evaluate direct medical costs per patient during IVF treatments, including ICSI as an optional step of the procedure. Clinical and economic outcomes were analyzed for two different ovarian stimulation approaches (rFSH + myo-Ins; rFSH only) up to three treatment cycles [first cycle involved, by definition, a fresh embryo transfer (ET)].

**Figure 1:**
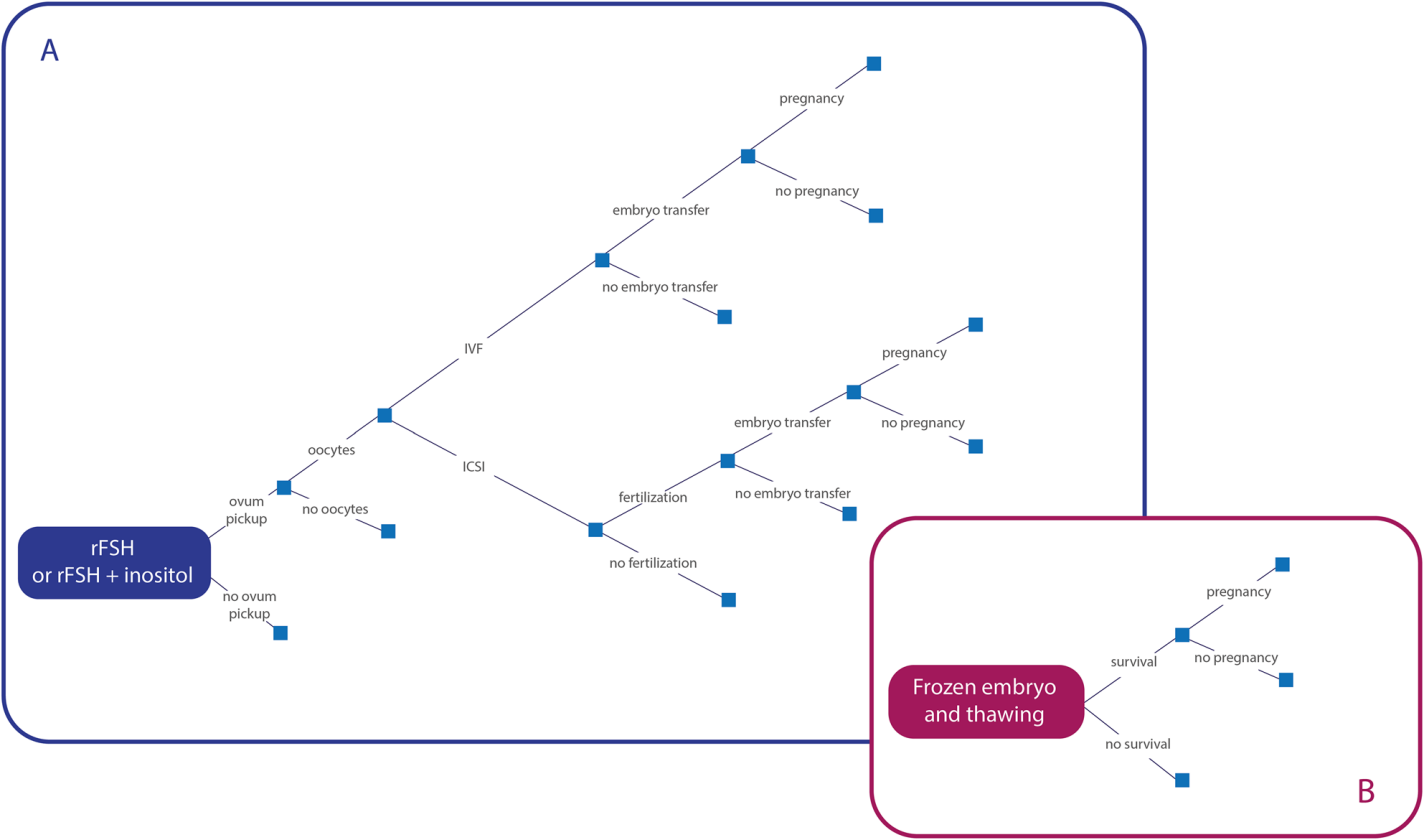
model structure. General architecture of the simulation model presenting the possible pathways of the (**A**) "fresh cycle" (complete ART cycle) and (**B**) the "frozen cycle".

Figure 1A provides a simplified representation of the fresh cycle simulation models, with the inset Fig. 1B representing the structure of the frozen cycle model.

The transition from one step in infertility treatment to another one (for instance, from oocyte retrieval to ET) is associated with a specific probability, referred as *transition probability* in this model. Estimates of transition probabilities were derived from the literature, clinical reports, and expert opinions. The transition probabilities used in the model are given in Tables 1 and 2, using a uniform distribution between minimum and maximum values. The costs associated with ART protocols are reported in Table 3 and are representative of the Italian situation.

**Table 1:** Transition probability of rFSH + myo-Ins versus only rFSH COH.

	rFSH + myo-Ins		rFSH	
	Min (%)	Max (%)	Min (%)	Max (%)
**Fresh cycle**				
Oocyte pick up	70	80	70	80
Oocytes after pick up	85	95	80	90
IVF versus ICSI	10	50	10	50
Fertilization after IVF	35	75	30	70
Embryo transfer after IVF fertilization	75	95	70	90
Pregnancy after IFV embryo transfer	20	45	20	45
Fertilization after ICSI	85	100	80	100
Embryo transfer after ICSI fertilization	85	100	80	100
Pregnancy after ICSI embryo transfer	20	45	20	45
**Frozen cycle**				
Survival frozen embryo	90	100	90	100
Clinical pregnancy after survived embryo	20	45	20	45

Table of transition probability associated with a rFSH + myo-Ins strategy in ART. Percentage ranges referred to the parameters in the table were obtained from the collection of published data^12,16,17^ and clinical experience. Reported numbers indicate the minimum and the maximum value recorded.

**Table 2:** Transition probabilities up to 3 IVF cycles.

	Min (%)	Max (%)
**Up to 3 cycles sequence**		
Initiating 2d cycle after failure 1st cycle	70	100
Fresh cycle at 2d cycle versus frozen cycle	40	60
Initiating 3rd cycle after failure 2d cycle	30	60
Fresh cycle at 3rd cycle versus frozen cycle	20	30

Table of transition probabilities. Percentage ranges referred to the parameters in the table represent the transition probabilities of combining up to 3 cycles and were obtained from the collection of published data^12,16,17^ and clinical experience. Reported numbers indicate the minimum and the maximum value recorded.

**Table 3:** Cost table.

	Min (€)	Max (€)
rFSH stimulation treatment	1000	2000
rFSH + inositol stimulation treatment	900	1900
Other hormons (gnRHa, hCG)	40	360
Ultrasounds	90	200
Consultations	60	200
Oocyte pickup	1000	1000
Ultrasounds	30	
**Fees**		
**IVF**		
Laboratory	300	600
**Fresh embryo transfer IVF**		
Catheter + disposables	100	200
OHSS treatment	90	150
ICSI	500	700
Laboratory	300	700
**Embryo transfer ICSI**		
Catheter + disposables	100	200
Frozen embryo and thawing	150	500
**Frozen embryo transfer**		
Catheter + disposables	100	200

The cost ranges referred to the parameters in the table represent each step of the IVF procedures, and were obtained from the collection of published data^12,18,19^ and clinical experience. Reported numbers indicate the minimum and the maximum value recorded.

The outcomes were defined as an ongoing pregnancy at 12 gestational weeks (confirmed by ultrasound scanning) for both fresh and frozen-thawed ET, and as the overall costs to achieve pregnancy through IVF with rFSH stimulation, either with or without associated oral myo-Ins supplementation.

Based on the most recent data about the topic^10^, we considered that each patient underwent treatment with 4 g myo-Ins per day, starting three months before COH and for the entire duration of the stimulation.

### Statistical analysis

A Monte Carlo technique was used to randomly generate the dispersion of outcomes at each stage, to allow determination of standard deviations and other distribution parameters for the model outcomes (total cost and overall pregnancy rate). By ascertaining the standard deviations, the presence of statistically significant differences between the outcomes can be determined.

For the cost effectiveness analysis, a virtual cohort of patients per group was used for the computer simulation of the ART program, using 100,000 Monte Carlo experiments to represent a probabilistic sensitivity analysis.

Since this is a cost-effectiveness analysis based on a computational simulation, a formal Institutional Review Board approval was not required.

## Results

### Cost of IVF procedure

After a maximum of three transfers, stimulation with rFSH and myo-Ins resulted in significantly lower total costs for the IVF procedure, compared with standard stimulation with rFSH (€ 4874 ± 2140 vs. 4986 ± 2165, *p* < 0.0001).

### Rate of ongoing pregnancies

The overall rate of ongoing pregnancies was significantly higher in the case of stimulation with rFSH + myo-Ins (0.38 ± 0.04), compared to stimulation with rFSH only (0.36 ± 0.06, *p* < 0.0001).

### Overall cost for successful pregnancy

The costs per successful pregnancy were significantly lower in the case of stimulation with rFSH + myo-Ins. From the Italian Health System perspective, the mean cost per pregnancy was € 13,001 ± 5898 when ovarian stimulation was carried out with rFSH and myo-Ins, compared to € 14,148 ± 6406 in the case of standard stimulation with rFSH (*p* < 0.0001).

## Discussion

The results of the Monte Carlo simulation suggest that the oral supplementation with myo-Ins during COH associated with rFSH in women with PCOS can represent a better cost-effective strategy for the Italian national health system, compared to ART protocols with standard gonadotropin stimulation. Even if preliminary, these results may indicate that by expanding the data available in literature with a virtual PCOS population, the advantages of myo-Ins administration could be considered a valuable approach in IVF procedures and deserve to be further investigated in the future and confirmed in clinical scenarios.

Usually, ART involves the manipulation of both male and female gametes to maximize the chances to achieve pregnancy^20^. IVF is the most frequently adopted ART and may include the injection of a single sperm directly into an egg (ICSI), depending on the causes of infertility^21,22^. In the last decade IVF evolved and improved, increasing the chances of success and the delivery rate^23,24^.

Like all ARTs, IVF is preceded by COH to yield a group of mature oocytes. Depending on the situation, the transfer of the best embryo(s) is carried out soon after the fertilization. In case of risk related to embryo transfer after COH, or in case of surplus of high-quality embryos, cryopreservation is a feasible option^25^. Hence, the overall analysis of IVF protocols should include both COH and the possibility of cryopreservation.

Several treatments have been evaluated to perform COH procedure in IVF, which may be properly indicated according to the cause of infertility^26^. Accumulating evidence suggests an effective control of ovarian stimulating with synthetic FSH, or rFSH^27^, but also a significant improvement of COH procedures with the association of myo-Ins administration^14^. myo-Ins is a pivotal molecule in human reproduction. As second messenger of FSH in the granulosa cells and important regulator of follicular microenvironment, it sustains the selection of the dominant follicle during the oogenesis^28,29^ favoring oocyte development^30^. Several studies further demonstrated a fundamental role of myo-Ins in restoring altered ovarian physiology, especially in women with polycystic ovary syndrome (PCOS). Indeed, they often exhibit anovulation and infertility issues^31^. Since these women may suffer from depletion of ovarian myo-Ins^32^, oral supplementation with this molecule significantly increases the regularity of the menstrual cycle and the ovulation rate^33^. Specifically, myo-Ins treatment restores ovulation in women with PCOS undergoing ART procedures^34^. Moreover, it reduces the number of degenerated oocytes, ameliorates the embryo quality, thus increasing the number of transferred embryos^16,28^, and finally enhances fertilization and pregnancy rates^30,35,36^. By boosting the ovarian sensitivity to FSH, oral myo-Ins administration lowers the units of gonadotropin and days of stimulation required in the COH protocols^12,37,38^, thus playing a positive role on ART outcomes^35,39^.

On these premises, we designed the present study to assess whether the administration of myo-Ins during ART protocols may be considered a cost-effective strategy, using the Italian Health System as reference model in order to reflect a real-world scenario. Since IVF procedures involve numerous steps (i.e.: ovarian stimulation, oocyte retrieval, fertilization, embryo transfer, and luteal support) and often multiple cycles to achieve success in terms of ongoing pregnancy and live birth, data from clinical trials are insufficient to provide useful cost-effectiveness information. Hence, computer-simulated clinical and economic models are well-suited options to perform pharmacoeconomic evaluations^19^. A model is a mathematical formula linking different variables to generate results relevant to a given environment, such as local medical practices. Results generated by modelling approaches thus provide unique information on the expected effectiveness, overall costs, and cost effectiveness of a set of ART cycles for assisting clinical decision making, as well as resource allocation decisions. Specifically, in the present analysis we used a computational model to compare the effectiveness and the costs of IVF protocols with two different COH strategies: standard stimulation with rFSH and treatment with rFSH associated with oral myo-Ins administration. We performed 100,000 Monte Carlo simulations of IVF cycles—with ICSI as an optional step—accounting for all the stages of the IVF procedure. Such choice is in line with the average number of procedures carried out in Italy every year^9^.

As treatments with myo-Ins reduce the dose of gonadotropins and length of stimulation required during COH^12,14^, the results from the simulation confirm that the association of myo-Ins and rFSH could significantly reduce the overall costs of the ART procedure, compared to the standard stimulation protocol with rFSH only. In detail, the mean cost per successful pregnancy has been calculated as € 13,001 (rFSH + myo-Ins) versus € 14,148 (rFSH). Since the Italian national health system covers the costs of rFSH for both private and public ART procedures^8^, the administration of myo-Ins during COH with rFSH may have a positive and tangible impact on the overall cost-effectiveness of the ART procedure.

Furthermore, the simulation demonstrates that the association of rFSH and myo-Ins improves the efficacy of the IVF procedure (either with or without ICSI) after a maximum of three cycles, with an estimated increase in the rate of ongoing pregnancies at 12 gestational weeks, compared with the standard rFSH stimulation. These data strengthen the evidence regarding the potential beneficial role of myo-Ins during ART treatments to sustain fertilization and pregnancy achievement, starting from the phase of ovarian stimulation^30,40^. Indeed, oral myo-Ins supplementation optimizes FSH signaling, providing proper follicular development and estradiol levels, synchronized with the day of the ovulation trigger.

As previously reported, better FSH signaling translates into lower amounts of exogenous gonadotropins necessary to achieve adequate oocyte maturation during COH. The use of the correct dosage of rFSH in ART procedures is of paramount importance: indeed, the quality of oocytes significantly worsens when FSH exceeds the amount required for the correct ovarian stimulation^41^. In this scenario, myo-Ins may play a pivotal role not only to optimize the COH procedures with rFSH, but also to reduce the number of cancelled cycled due to the risk of OHSS^42^, a rare yet extremely serious complication of ART procedures arising from the excessive ovarian stimulation with gonadotropins^43^. The overall higher efficacy of IVF protocols, associated with myo-Ins administration^44^, could also lead to lower psychological impact for women undergoing IVF.

myo-Ins is a pivotal factor in the ovarian physiology, and the presence of correct amounts of this molecule allows to correctly complete the foliculogenesis and the fertilization phases^28^. The importance of balancing appropriate levels of myo-Ins is particularly evident in PCOS context, where oral myo-Ins supplementation has been found to improve both metabolic and endocrine parameters^31,45^, regularizing the ovulatory menstrual cycles and ameliorating the IVF outcomes^46^. Additionally, several authors demonstrated that myo-Ins dietary supplementation is a natural and safety approach for women seeking pregnancy, on the contrary, this molecule sustain the prevention of anomalies in the fetus such as neural tube defects, spina bifida, and macrosomia^47–50^.

Several other stimulations procedures based on the use of gonadotropin or different drugs are currently adopted in ART programs, such as recombinant FSH and LH, urinary FSH, menopausal gonadotropins, pulsatile gonadotropin releasing hormone (GnRH), and also clomiphene citrate, letrozole, kisspeptin agonists and androgens^51^. Among these, various treatments have been investigated and compared, but quite often the cost-effectiveness results are not fully clear and thus do not allow to draw firm conclusions^52^. Considering these elements, the model described in the present study was designed to compare COH with rFSH plus myo-Ins against a COH with the only rFSH. The present model aims to investigate the potential advantages derived from the addition of myo-Ins to rFSH based COH, in term of cost-effectiveness for the Italian Health System.

Of note, some limitations must be considered for a proper data interpretation. The computational model adopted to simulate IVF cycles should be confirmed by more clinical data, in order to support the results from this investigation. This is specifically important, considering the difficulty to find available and informative data in this regard. Moreover, we chose to compare two of the most diffused protocol for COH, with rFSH only and rFSH + myo-Ins because myo-Ins was found to act as FSH second messenger, although we acknowledge that other stimulation protocols should be considered and evaluated in the future, especially cheaper option for ovarian stimulation such as highly purified human menopausal gonadotrophin (HP-hMG). In addition, it might be considered that our virtual population is a projection of the PCOS subjects commonly observed in IVF procedures and is not representative also of different patients exhibiting co-morbidities or obese women. The present simulation was based on the Italian Health System in order to reflect a real-world scenario and, for this reason, may not be directly replicable to other national health system without a proper evaluation data and costs of the correspondent country.

In addition, we should consider that oral absorption of myo-Ins could be affected by several factors, including the formulation of the supplement^53^, the dose of the compound, the presence of additional carrier molecules, and/or gastrointestinal pathologies^54^. In this scenario, available evidence from in vitro studies and clinical trials suggests that up to 30% of patients treated with myo-Ins poorly respond to the intervention because of impaired intestinal absorption. Such condition was referred as *inositol resistance*. In this context, active peptides from α-lactalbumin (α-LA) proved to enhance the intestinal bioavailability of myo-Ins by increasing the permeability of the tight junctions of the human intestinal epithelium^54^. Clinical data demonstrated that α-LA allows to overcome inositol resistance in women with PCOS, restoring ovulation in over 85% of resistant subjects, who failed to respond to the treatment with the sole myo-Ins^55^. This result was further confirmed with a multicenter clinical trial, where the association of myo-Ins and α-LA significantly improved the conditions of women with PCOS and insulin resistance^56^. All these data indicate that co-administration with α-LA is recommended to enhance the potential benefits of myo-Ins supplementation.

## Conclusions

The present study suggests the potential benefits of oral myo-Ins supplementation during ART procedures in terms of cost/effectiveness when investigated in the Italian national health system. Through a computer simulation, we compared IVF protocols performed in PCOS women with two COH approaches (stimulation with rFSH versus stimulation with rFSH associated to myo-Ins supplementation), demonstrating that myo-Ins supplementation improves the success rate for IVF, and reduces the costs per single pregnancy and the overall costs of IVF protocols. Based on these preliminary evidence, ovarian stimulation with rFSH and myo-Ins in women affected by PCOS, could be considered a dominant strategy with respect to the standard stimulation with rFSH only. While further analyses are necessary to confirm these data on a larger population and in a greater dataset obtained from clinical practice, these findings may translate in a reduced impact on a national health system like the Italian one, which covers the costs of the gonadotropins needed for the ovarian stimulation during IVF treatments.

## Data Availability

Data will be made available to the editors of the journal for review or query upon request. If data are required for any purpose, please contact Dr. Michele Russo. Email address: m.russo@lolipharma.it.
